# Nocturnal Sleep Related with Metabolic Markers in End-Stage Renal Disease Patients Receiving Hemodialysis

**DOI:** 10.4306/pi.2009.6.1.34

**Published:** 2009-03-31

**Authors:** Jung Hie Lee, Seong Jae Kim, Hae Hyuk Jung

**Affiliations:** 1 Department of Psychiatry, Kangwon National University College of Medicine, Chuncheon, Korea.; 2 Department of Neuropsychiatry, Hyosung General Hospital, Cheongju, Korea.; 3 Department of Internal Medicine, Kangwon National University College of Medicine, Chuncheon, Korea.

**Keywords:** End-stage renal disease, Nocturnal sleep, Uric acid, C-reactive protein, Sleep breathing disorder

## Abstract

**Objective:**

It has been suggested that oxidative stress and inflammation are associated with the pathophysiology of sleep disorders in end-stage renal disease (ESRD) patients. We examined the relationship of the sleep variables reflecting sleep breathing disorder and limb movements during sleep with the clinical variables reflecting the metabolic abnormalities in ESRD patients receiving hemodialysis.

**Methods:**

Nocturnal polysomnography was conducted in 30 ESRD patients (21 men, 9 women), who were receiving hemodialysis. Blood was sampled before hemodialysis for each patient in order to measure uric acid (UA), C-reactive protein (CRP), and interleukin-6 (IL-6).

**Results:**

UA was correlated positively with the total sleep time (TST)(r=0.407) and negatively with the apnea-hypopnea index (AHI) and oxygen desaturation index (ODI)(r=-0.377, -405).

**Conclusion:**

CRP was positively correlated with the limb movement index (LMI)(r=0.401). Our study showed that increased UA was related to decreased respiratory disturbance during sleep in ESRD patients receiving hemodialysis.

## Introduction

Sleep problems are very common in patients with end-stage renal disease (ESRD), as more than 80% of them complain of difficulty in maintaining sleep, restless legs, and daytime sleepiness.^1^ These symptoms could be related to primary sleep disorders such as sleep apnea (SA), periodic limb movements in sleep (PLMS), or restless legs syndrome (RLS). The prevalence of SA in ESRD patients was reported to range from 50 to 70%, which is more than 10 times that of the general population; the prevalence of PLMS also ranged from 30 to 69%.^2^-^4^ Many factors affect sleep disturbances in ESRD patients: psychological factors such as depression, anxiety and stress; metabolic factors related to renal failure such as uremia and anemia; and hemodialysis (HD)-related factors such as drugs and electrolyte imbalance.^5^

Recently, the increased prevalence of oxidative stress and inflammation in patients with moderate to severe chronic kidney disease was reported.^6^ Oxidative stress and acute-phase inflammation may be critical to the pathophysiology of various clinical symptoms related to ESRD, particularly in the cardiovascular complications that raise the mortality rate of ESRD patients.

Similarly, SA has been recognized as an oxidative stress disorder. It has also been reported that patients with SA have elevated inflammatory markers such as interleukin-6 (IL-6), tumor necrosis factor (TNF)-α and C-reactive protein (CRP), and have a higher risk of cardiovascular disease than the general population.^7^ That is, oxidative stress and inflam mation may be associated with the pathophysiology of SA in ESRD. Although some studies have investigated sleep disorders in terms of the metabolic abnormalities in ESRD patients,^2^,^8^ few have investigated them in terms of oxidative stress and inflammation. Among the metabolic variables related with ESRD, uric acid (UA) has not been a significant research topic in previous studies of sleep disorders in ESRD patients, even though it has a strong antioxidant capacity.^9^

We aimed to examine the relationship of the nocturnal sleep characteristics linked to sleep breathing disorder (SBD) or limb movements during sleep with the levels of UA, IL-6 and CRP, which act as metabolic markers reflecting the antioxidant capacity or inflammation in ESRD patients receiving hemodialysis.

## Methods

### Subjects

We studied ESRD patients who were receiving hemodialysis at Kangwon National University Hospital from October 2004 until December 2005.

According to the guidelines of the National Foundation-Dialysis Outcome Quality Initiative (NKF-DOQI) in United States,^10^ ESRD patients were enrolled only when their Kt/Vurea (Kt/V) level was above 1.2, reflecting the stability of hemodialysis. We excluded subjects with a history of psychiatric disease, present psychiatric illness requiring treatment, organic brain disease, history of heart failure and chronic pulmonary disease, or acute medical illnesses such as pneumonia, urinary tract infection, or enteritis, and those taking medication affecting sleep. Nine subjects who refused to participate in the study were also excluded.

For 30 ESRD patients, the Epworth Sleepiness Scale (ESS)^11^ and Sleep Disorders Questionnaire (SDQ)^12^ were administered. SDQ was completed for only 16 patients. For the remaining 14 patients, SA subscale was administered instead of SDQ because of the difficulty completing the latter. No subject was found to have a major depressive disorder according to the Diagnostic and Statistical Manual of Mental Disorders, fourth edition (DSM-IV),^13^ criteria and based on a clinical interview conducted by a psychiatrist. The information on the history of smoking and drinking alcohol, current medication, duration of hemodialysis, and illness preceding ESRD was gathered through medical records, and the body mass index (BMI) was also obtained for each subject. Among 30 patients, none had a diagnosis of alcohol abuse or dependence, and five were current smokers (Mean pack years±SD=35±15.45). Four patients stopped taking sleeping pills for 2 weeks prior to the sleep study. The study protocol was approved by our institutional review board, and written informed consent was obtained from each enrolled patient.

### Sleep studies

All subjects were studied by one-night laboratory-based polysomnography (PSG). Considering the effect of hemodialysis on sleep, the PSG (Embla S7000, Medicaresystem, New York, USA) was conducted according to each subject's habitual sleep time one day after hemodialysis. PSG included electroencephalogram (C3-A2, C4-A1, O1-A2, O2-A2 by international 10-20 system), chin electromyography (EMG), electrooculogram, electrocardiogram, snoring, respiratory effort using piezoelectric belts over the chest and abdomen, and airflow through the nose and mouth using a thermistor. We also recorded bilateral surface EMGs on the legs (with electrodes placed over the anterior tibialis muscles). We monitored oxygen saturation by pulse oximetry. A polysomnographic technologist, who had an interrater reliability of more than 90% with a psychiatrist certified by the American Board of Sleep Medicine, manually scored all of the recordings for the sleep stages, limb movements, and respiratory events by standard techniques.^13^ The oxygen desaturation index (ODI) was defined as the mean number of events per hour in which the oxygen saturation level was decreased by more than 3% during sleep. Sleep apnea syndrome (SAS) was diagnosed when the apnea-hypopnea index (AHI) was 5 or greater, and obstructive sleep apnea-hypopnea syndrome (OSAHS) when the sum of the obstructive apnea index (OAI) and hypopnea index (HI) was 5 or greater.^15^ The limb movement index (LMI) was defined as the mean number of limb movements per hour. Limb movements immediately following respiratory events were not scored. Periodic limb movements (PLMs) were defined when more than 4 consecutive limb movements with a duration of 0.5-5 seconds were observed every 5-90 seconds. The periodic limb movement index (PLMI) was defined as the mean number of periodic limb movements per hour. A diagnosis of PLMS was made when the PLMI was 5 or greater.^15^

### Laboratory studies

Blood was sampled for each subject before hemodialysis to measure the UA, CRP, and IL-6 levels. UA was measured by the enzymatic method (Denka, Tokyo, Japan) with a TBA-200FR Chemical analyzer. CRP was analyzed by the latex agglutination quantitative method (Denka, Tokyo, Japan) with a TBA-200FR device (Toshiba, Tokyo, Japan). IL-6 was measured by enzyme-linked immunosorbent assay (ELISA)(Endogen, Woburn, USA). The assay ranged from 15-2,450 pg/mL; the intraassay and interassay coefficients of variation were below 10%. The detection limit of the IL-6 ELISA was 15-20 pg/mL. The indirect indices of protein intake, serum albumin, and normalized protein catabolic rate (nPCR){normalized to body weight derived from the urea distribution space (Vurea/ 0.58)} were obtained. For all variables, the values were the means of 3 measurements, except for IL-6, which was measured once. The IL-6 level was obtained for only 23 of 30 ESRD patients, because it was not detected in the other 7 patients.

### Statistical analysis

Spearman correlation analyses were used to examine the relationship between the sleep variables and clinical variables of UA, IL-6, and CRP. A Pearson correlation analysis on nPCR and UA was conducted, considering that nPCR is a marker reflecting the nutrition state in ESRD patients. All statistical analyses were performed using Statistical Package for the Social Sciences (SPSS) version 11.5, and p values below 0.05 indicated statistical significance.

## Results

Among 30 patients, 15 (50.0%) had diabetes, 8 (26.7%) glomerulonephritis, 3 (10.0%) hypertension, and 4 (13.3%) unknown diseases as the main causes of ESRD. There was no significant difference in any of the sleep variables among the various groups of patients with different causes of ESRD (Kruskal-Wallis test, p>0.05). The prescribed drugs were antilipemic agents for 7 patients (23.3%), ACE inhibitors for one (0.03%), beta-blockers for 13 (43.3%), intravenous iron for 6 (20.0%), and statins for 7 (23.3%). The accompanying illnesses were ischemic heart disease in 2 patients (6.6%) and left-ventricle hypertrophy in 19 (63.3%), as well as diabetes in 15 (50%). The demographic/ clinical characteristics and Nocturnal polysomnography (NPSG) results are presented in Table 1 and 2, respectively. There was no significant difference in the UA, IL-6 and CRP levels between the smoking and non-smoking groups (Mann-Whitney test, p>0.05).

Among 30 patients with ESRD, 28 (93%) were diagnosed as having SAS or PLMS; 25 (83.3%) had a diagnosis of SAS, 18 (60%) OSAHS, and 18 (60%) PLMS. Fifteen patients (50%) had both SAS and PLMS, 13 patients (43.3%) had both OSAHS and PLMS, and 2 patients had diagnoses other than the primary sleep disorder. Upon reviewing the questions related to RLS in the SDQ (Q12. restless legs in falling asleep, Q31. restless legs disturb sleep) for 16 patients who completed it, RLS was diagnosed in 5 patients (31%).

Among the metabolic markers in 30 HD patients with ESRD, UA was positively correlated with the total sleep time (TST)(r=0.407)(Table 3) and negatively with AHI and ODI (r=-0.377, -0.405), and CRP was positively correlated with LMI (r=0.401)(Table 4). UA was also significantly correlated with nPCR (r=0.492, p<0.05).

## Discussion

In this study, the proportions of patients with sleep disorders and SAS among 30 ESRD patients were 93.3% and 83.3% respectively, which were higher than those of previous studies (50-70%).^2^,^3^ Parker et al.^2^ showed that 23 among 46 HD patients (50%) had an AHI above 5, a result that seems to differ from ours, for they excluded those subjects with SAS, PLMS, or RLS by means of a structured interview. Kimmel et al.^3^ reported that the proportion of OSAHS patients among those ESRD patients with SAS was 56% (9/16), which is lower than 72% (18/25) in the present study: 60% of our ESRD patients were diagnosed with PLMS. It has been reported that the incidence of PLMS in ESRD patients is 30-70% in general.^2^,^4^ In our study, it is notable that 19 patients (50%) had diagnoses of both SAS and PLMS. Previous studies have reported either SAS or PLMS separately, except for the study of Parker et al.^2^ where 12 (26%) of 46 ESRD patients had both an AHI and LMI greater than 5. In our study, 5 (31%) of 16 ESRD patients who completed the SDQ complained of RLS symptoms. In previous studies, RLS has been variously reported as affecting 7-60% of ESRD patients. Rijsman et al.^16^ reported that 58.3% of ESRD patients had RLS, and PLMS was found in 90% of those with RLS. The RLS with PLMS seems to be clinically important in ESRD patients, since it worsens the quality of life.^16^

Increased UA was related to increased total sleep time (TST) and to decreased AHI and ODI in our study. Hsu et al.^21^ recently suggested a positive effect of UA related to its antioxidant properties, predicting higher mortality in HD patients with lower UA levels. However, UA increases in oxidative stress situations such as hypertension, cardiovascular disease, and obstructive sleep apnea (OSA).^17^-^20^ So, it remains controversial whether serum UA actually has antioxidant properties.

Both OSA^19^ and ESRD have been considered as oxidative stress disorders.^6^ Although the mechanism related to increased oxidative stress could be explained by various factors in ESRD patients, the major cause would be the retention of oxidized solute by the loss of renal function. Supporting our result that increased UA was related to an improvement in SBD, UA has been suggested to have antioxidant properties in several studies. Hasegawa et al.^9^ suggested that UA plays an important role as an antioxidant in ESRD patients, and Christou et al.^22^ reported that UA's antioxidant capacity was significantly decreased in OSA patients with an AHI of more than 20. Our previous study also found that increased ODI in SAS was related to decreased total antioxidant status (TAS) level, reflecting the antioxidant capacity in ESRD patients.^23^ In ESRD patients, the association of OSA with a deficit of antioxidant capacity may imply that the oxidative stress reaction is increased due to the reduced level of cellular reductants under a hypoxemic state such as OSA, or the reduced antioxidant capacity results in the occurrence or aggravation of OSA. Our study showed that UA levels were significantly correlated with the nPCR (r=0.492), reflecting the nutritional state of proteins. Söreide et al.^8^ reported that the intake of branch-chain amino acids (BCAAs) in ESRD patients was associated with respiratory stimulation during sleep. Malnutrition would also be associated with increased oxidative stress, thus explaining the mechanism underlying the relationship between protein nutrition and respiration during sleep, although this explanation is not conclusive.^24^ Therefore, the improvement of SBD may be due to the antioxidant properties of UA, reflecting protein nutrition.

As the CRP level increased, the LMI increased in our study, and there was no sleep parameter correlated with IL-6. In ESRD patients, although it was reported that sleepiness was induced by the abnormal production of TNF, a substance with somnogenic properties,^25^ the relationship of the sleep variables with inflammatory markers such as CRP and IL-6 has not yet been studied. ESRD is recognized as a chronic inflammatory disease, because these inflammatory markers are higher in ESRD patients.^6^ On the other hand, it is known that the metabolic abnormality of dopamine can cause an inflammatory reaction,^26^ and impaired dopaminergic neurotransmission is a major factor in the pathogenesis of RLS,^27^ which is often accompanied by increased limb movement (LM) during sleep. Accordingly, increased CRP would have been correlated with increased LM. However, IL-6, another inflammatory marker, was not correlated with LM in our study. This could be explained by the instability of the measurement of IL-6, since IL-6 was not detected in 7 patients, and the IL-6 data were measured only once. Therefore, the relationship between inflammatory markers and LM in ESRD patients cannot be definitively determined from our results, and further studies will be necessary.

The limitations of our study are as follows. First, comorbid medical diseases such as hypertension and diabetes mellitus were not excluded, since these are relatively common in ESRD patients. Since it was also very difficult to exclude patients taking some form of medication (except hypnotics), these substances may have affected nocturnal sleep in our study. Second, our results are not conclusive, since confounding variables such as age, sex and BMI, which affect AHI and LMI, were not controlled. Third, we did not conduct multiple sleep studies to reduce the first-night effect and ESRD patients with possible primary sleep disorders were not excluded from our study, resulting in the presence of extreme values of the nocturnal sleep variables.

However, a limited number of studies on the relationship between sleep disorder and metabolic markers in ESRD patients have been reported. In Asian countries, there has only been one study using the sleep questionnaire without conducting NPSG. Considering the recent findings that ESRD is recognized as an oxidative stress disease or an inflammatory disease, it is meaningful that UA was significantly related to decreased severity of SBD, and CRP was related to increased severity of LM in our study. These findings suggest that UA or CRP levels reflect certain sleep disorders in ESRD patients. Further studies on the relationship of these markers to the pathophysiology of sleep disorders are necessary.

## Figures and Tables

**TABLE 1:**
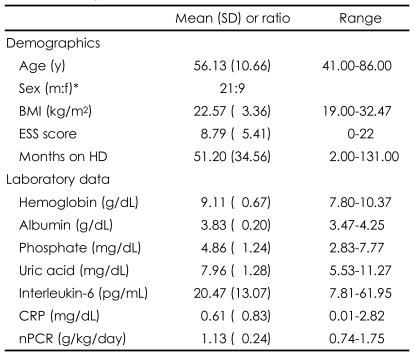
Demographic and clinical characteristics of end-stage renal disease (ESRD) patients receiving hemodialysis (N=30)

BMI: body mass index, ESS: Epworth Sleepiness Scale, HD: hemodialysis, CRP: C-reactive protein, nPCR: normalized protein catabolic rate

**TABLE 2:**
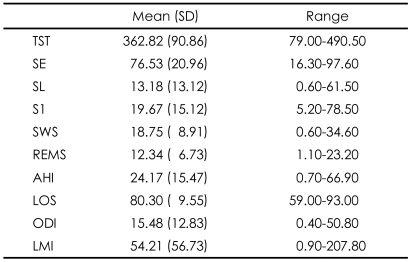
Nocturnal sleep characteristics in ESRD patients receiving hemodialysis (N=30)

ESRD: end-stage renal disease, TST: total sleep time (min), SE: sleep efficiency (%), SL: sleep latency (min), S1: stage 1 sleep (%), SWS: slow wave sleep (%), REMS: rapid eye movement sleep (%), AHI: apnea-hypopnea index (n/hr), LOS: lowest oxygen saturation (%), ODI: oxygen desaturation index (n/hr), LMI: limb movement index (n/hr)

**TABLE 3:**
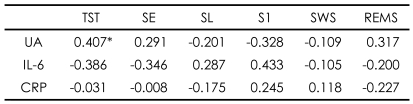
Correlations of sleep architecture variables with metabolic markers ESRD patients receiving hemodialysis (N=30)

^*^p<0.05 (Spearman correlation analysis). ESRD: end-stage renal disease, TST: total sleep time (min), SE: sleep efficiency (%), SL: sleep latency (min), S1: stage 1 sleep (%), SWS: slow wave sleep (%), REMS: rapid eye movement sleep (%), UA: uric acid, IL-6: interleukin-6, CRP: C-reactive protein

**TABLE 4:**
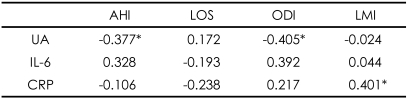
Correlations between the variables related with sleep breathing disorder and limb movements and metabolic markers in ESRD patients receiving hemodialysis (N=30)

^*^p<0.05 (Spearman correlation analysis). AHI: apnea-hypopnea index (n/hr), LOS: lowest oxygen saturation (%), ODI: oxygen desaturation index (n/hr), LMI: limb movement index (n/hr), UA: uric acid, IL-6: interleukin-6, CRP: C-reactive protein
